# Recovery of mobility function and life-space mobility after ischemic stroke: the MOBITEC-Stroke study protocol

**DOI:** 10.1186/s12883-020-01920-z

**Published:** 2020-09-16

**Authors:** R. Rössler, S. A. Bridenbaugh, S. T. Engelter, R. Weibel, D. Infanger, E. Giannouli, A. Sofios, L. Iendra, E. Portegijs, T. Rantanen, L. Streese, H. Hanssen, R. Roth, A. Schmidt-Trucksäss, N. Peters, T. Hinrichs

**Affiliations:** 1 Division of Sports and Exercise Medicine, Department of Sport, Exercise, and Health, University of Basel, Birsstrasse 320 B, 4052 Basel, Switzerland; 2 Basel Mobility Center, University Department of Geriatric Medicine Felix Platter, Basel, Switzerland; 3 Neurology und Neurorehabilitation, University Department of Geriatric Medicine Felix Platter, Basel, Switzerland; 4 Department of Neurology & Stroke Center, University Hospital Basel, University of Basel, Basel, Switzerland; 5 Department of Geography, University of Zurich, Zurich, Switzerland; 6 Faculty of Sport and Health Sciences & Gerontology Research Center, University of Jyvaskyla, Jyvaskyla, Finland; 7 Neurology & Stroke Center, Klinik Hirslanden, Zurich, Switzerland

**Keywords:** Aging, Mobility limitation, Walking speed, Quantitative gait analysis, Spatial behaviour, Quality of life, Accelerometers, GPS, Cohort study

## Abstract

**Background:**

Stroke is a major cause of disability and stroke incidence increases with age. Stroke frequently results in permanent limitations of mobility, and, consequently, the need for the help of others in activities of daily living. In order to optimize rehabilitative efforts and their functional outcomes, detailed knowledge of the functional recovery process, regarding mobility, is needed. Objectives of the MOBITEC-Stroke study are: 1.) To characterize mobility, including lower extremity physical function (LEPF) and life space (the geospatial extent of all of a person’s movements), and changes in mobility within the first year after stroke. 2.) To identify and characterize subgroups with different mobility trajectories. 3.) To evaluate whether changes in LEPF are associated with changes in life-space. 4.) To evaluate participants’ reasons for going outdoors, transportation use, and assistance needed for outdoor movement.

**Methods:**

Patients with incident first stroke who live in their own homes (target *N* = 59, based on sample size calculation) will be included in this cohort study. At 3, 6, 9, and 12 months after stroke a battery of mobility tests will be performed at the study centre, including laboratory-based tests of balance and strength, and quantitative gait analysis. Life-space assessment (including 1-week GPS measurements) will be performed in participants’ real life. Semantic information on visited locations (reasons for going outdoors, transportation use, assistance needed) will be collected by using interactive digital maps. Linear mixed effects models will be used to model the trajectories of mobility measures for the total sample and for predefined subgroups. As an exploratory analysis, growth mixture models (GMMs) will be used to identify relevant subgroups with different trajectories. Linear mixed effect models will be used to test whether changes in LEPF parameters are associated with changes in life-space. Participants’ motivation for going outdoors, transportation use, and assistance needed for outdoor mobility will be analysed descriptively.

**Discussion:**

A comprehensive and detailed knowledge of recovery patterns will enable the planning of targeted and adaptively tailored rehabilitation measures. Information about patients’ reasons for outdoor mobility will provide the opportunity to define individualized and patient-oriented rehabilitation goals.

**Trial registration:**

ISRCTN85999967 (on 13 August 2020; retrospectively).

## Background

Stroke is among the leading causes of mortality and acquired long-term disability worldwide, its incidence increases with age [1–5]. If survived, stroke often results in permanent limitations of mobility [6]. “Mobility” has been defined comprehensively as “the ability to move oneself (either independently or by using assistive devices or transportation) within environments that expand from one’s home to the neighbourhood and to regions beyond” [7], (p. 444). Thus, measures that describe a person’s mobility include tests of “lower extremity physical function” (LEPF) (also referred to as “mobility function” (e.g., [8]) [9], and assessments of “life-space” [10]. So far, research on recovery of mobility after stroke has mainly focused on function [11–15].

Reduced LEPF after stroke, initially attributed to the brain lesion, leads to a vicious circle of sedentary behaviour [16], disuse muscle atrophy and weakness [17], fear of falling, and falls [18]. It also results in lower health-related quality of life [19], disability in basic activities of daily living (ADL) and self-care [20], and consequently need for personal assistance and institutional care [21]. So far, studies on LEPF in people after stroke mostly relied on questionnaires or simple functional tests, the latter often including (subjective) therapist ratings [11–13, 15, 22]. More sophisticated and precise measures of LEPF are, however, available, including instrumented assessment of strength, balance, and gait (e.g. with dynamometers, pressure sensitive walkways or body worn accelerometers), allowing the disentanglement of the exact components responsible for mobility limitation. In contrast to most functional tests, these measures can also account for the asymmetry, which is typical for stroke patients [23, 24]. Rehabilitative measures to restore LEPF in people after stroke include physical therapy, exercise (including training of balance, strength and gait), and the provision of adaptive devices [25–27]. It has previously been argued that there may be specific therapeutic windows during stroke recovery in which certain rehabilitative measures are likely to be most successful [28, 29]. Besides the difficulty of applying the correct rehabilitative measures at the right time, the patient’s adherence to the rehabilitative measures is crucial. It has been shown that adherence to rehabilitative measures in stroke patients already starts to decrease 6 weeks post-stroke and reaches its minimum 21 weeks post-stroke [30]. Lack of motivation has been found to be one of the main reasons for low adherence to exercise in stroke patients [31]. As a majority of people after stroke (75% of participants in a New Zealand study) consider “the ability to get out and about in the community” essential or very important shortly after returning to their own homes [32], regaining the ability to independently move within one’s environment (i.e. extending the life-space) might serve as a strong motivator for patients to adhere to their rehabilitation plans.

Life-space, the “spatial extent in which a person moves within a specified period” encompasses “the interaction between intrinsic capabilities of the person and the demands of the extrinsic environment” [33], (p. 155). In the general older population, restricted life-space is predictive of disability in ADL [34], frailty [35], falls and fractures [36], nursing home admission [37], and mortality [38]. Epidemiological studies usually rely on questionnaires to measure life-space (e.g., [10]), however, they are prone to recall bias and their geospatial resolution is low. Nowadays, Global Navigation Satellite Systems (GNSS), such as the Global Positioning System (GPS), offer the chance to objectively and much more precisely measure a person’s life-space [39, 40]. Additional semantic information, e.g. on the purpose of visiting a certain location, on the use of transportation, on the need for personal assistance or on environmental facilitators of mobility, can be collected by interactive digital maps [41–43]. Despite its relevance for personal health and social interaction, life-space after stroke has scarcely been examined: a single cross-sectional questionnaire-based Korean study of 34 people after stroke showed that life-space was positively associated with functional ambulation and independence [44]. Longitudinal studies assessing life-space and social participation of stroke patients repeatedly at clearly defined time periods after stroke and using objective measures of life-space mobility are missing.

In summary, in order to be able to design individualized rehabilitative measures for people after stroke and to maximize their effectiveness, knowledge of the details of stroke recovery (including timing and components) and of the association between LEPF and life-space during the recovery process is urgently needed. This will allow health care professionals to apply the correct rehabilitative measures at the right time and at the optimal dosage, and to estimate the degree of recovery that can be achieved. The additional knowledge of why people want to visit certain places and what enables them to get there will allow therapists to define individualized treatment goals with a focus on patient empowerment.

## Methods and design

### Objectives

This study has the following objectives:

1. To characterize mobility, including LEPF and life-space, and changes in mobility within the first year after stroke
2. To identify subgroups with different mobility trajectories and describe the patient characteristics for each subgroup
3. To evaluate whether changes in LEPF parameters are associated with changes in life-space
4. To evaluate reasons for going outdoors, transportation use and assistance needed

### Design

**Fig. 1:**
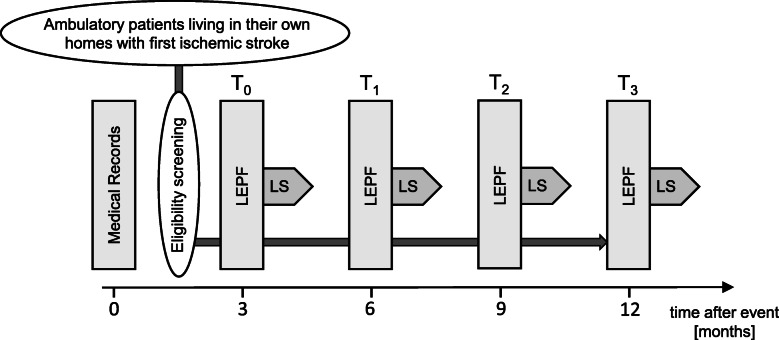
Study timeline. Laboratory assessments of lower extremity physical function (LEPF) and subsequent ambulatory 1-week assessments of life-space (LS) take place at 3, 6, 9 and 12 months after ischemic stroke

MOBITEC-Stroke (“Recovery of mobility function and life-space mobility after ischemic stroke”) is designed as a prospective cohort observational study. Clinical evaluation and follow-up as well as mobility measurements will be performed at four time points: 3 (T_0_), 6 (T_1_), 9 (T_2_), and 12 (T_3_) months after stroke. Furthermore, clinical information for the time of first admission to the Stroke Centre will be available for all subjects (i.e., shortly after the event). Each measurement will consist of a battery of tests conducted at the study centre (Basel Mobility Center, Department of Geriatric Medicine Felix Platter) as well as a subsequent 1-week measurement with wearable sensors around participants’ homes (Fig. 1).

### Target group, inclusion criteria and exclusion criteria

#### Target group

The study targets ambulatory patients after first ischemic stroke living in their own homes following stroke.

#### Inclusion criteria

The study includes female and male patients from 18 years of age with first ischemic stroke within the past 3 months. Participants must be able to communicate verbally with the study personnel; they have to be able to understand the study information and have to provide written informed consent. At least one of the following stroke-related symptoms potentially affecting gait and mobility must be present: lower limb paresis or ataxia; stance/gait ataxia (cerebellar or sensory); visual disturbance/field defect; central vestibular deficit; attentional deficit/neglect. Participants have to be able to get up from a chair and sit down without external help and to walk for a minimum of 20 m at their own pace, with or without pauses, with or without a walking aid, but without the physical assistance of another person (self-report).

#### Exclusion criteria

Persons who are community-dwelling, are unable to walk without assistance, are wheelchair bound or permanently bedridden (modified Rankin Scale, mRS > 3 points) [45, 46], and persons with presence of severe cognitive impairment (Montreal Cognitive Assessment (MoCA) score < 21 or, < 20 for persons with 12 years of education or less) [47, 48], an acute psychiatric disorder (e.g. severe depression), or an advanced terminal illness are excluded. Orthopaedic surgery of the lower extremities within the past year and on-going rehabilitation measures following an inpatient surgical procedure at the time of stroke are further exclusion criteria. Patients with major pre-stroke mobility limitations (major difficulties in walking or climbing stairs; self-report) are also excluded. The usage of centrally acting drugs will be documented but will not per se be an exclusion criterion.

#### Recruitment

Participants will be recruited through the Department of Neurology and Stroke Centre, University Hospital Basel and the Department of Geriatric Medicine Felix Platter. Eligible patients will be informed about the study and invited to participate.

### Measures

#### Participant characteristics

The following standardised information from first admission to the Stroke Centre (i.e., shortly after the event) will be available for all subjects: sex, age, date, and time of symptom onset or last proof of good health; date and time of arrival at hospital; National Institutes of Health (NIH) stroke scale [49] on admission and 24 h after admission; stroke localisation (brain region and vascular territory affected); first brain imaging type (CT/MRI) and result; etiology of ischemia; type of initial treatment; date of hospital discharge and discharge destination.

The following additional participant characteristics will be assessed by self-report at T_0_ (Table 1): years of education and pre-stroke social support (regular company when going outdoors and for errands) [50]. Body height and leg length will be measured by a trained assessor.

**Table 1:** Schedule of assessments

Assessment	Recruit-ment	T_**0**_ (3 months after stroke)	T_**1**_ (6 months after stroke)	T_**2**_ (9 months after stroke)	T_**3**_ (12 months after stroke)
**Participant characteristics**					
Cognition (MoCA)	x				
Body height		x			
Leg length		x			
Body weight		x	x	x	x
Years of education		x			
Social support (incl. Pre-stroke)		x	x	x	x
Financial hardship		x	x	x	x
Residential area		x	x	x	x
Housing situation		x	x	x	x
Living condition		x	x	x	x
Instrumental activities of daily living (IADLs)		x	x	x	x
Clinical-neurological examination		x			x
Stroke severity (NIHSS)		x			x
Level of functional independence (mRS)		x			x
Comorbidities (SCQ)		x			x
Depressive Symptoms (GDS-15)		x			x
Health-related quality of life (SS-QoL)		x			x
Use of centrally acting drugs		x			x
Vison problems		x			x
Hearing problems		x			x
**Mobility**					
Walking ability (incl. Pre-stroke)		x	x	x	x
Mobility limitations (incl. Pre-stroke)		x	x	x	x
Quantitative gait analysis (pressure sensitive walkway; portable sensors)		x	x	x	x
Lower limb muscle power (leg press)		x	x	x	x
Balance (force platform)		x	x	x	x
5 times sit-to-stand (on force platform)		x	x	x	x
Timed up-and-go test		x	x	x	x
Objective life-space (1-week GPS)		x	x	x	x
Self-reported life-space (UAB LSA)		x	x	x	x
Reasons for going outdoors, transportation use, need for assistance (digital map-based tool)		x	x	x	x
Availability and usage of a private car (incl. Pre-stroke)		x	x	x	x
**Further measures**					
Physical activity (1-week accelerometry)		x	x	x	x
Active Ageing (UJACAS)		x	x	x	x
Fall-history (3-month recall) (incl. Pre-stroke)		x	x	x	x
Fall-related self-efficacy (FES-I)		x	x	x	x
Retinal vessel analysis			x		x
Rehab measures		x	x	x	x
Medical events		x	x	x	x

*MoCA* Montreal Cognitive Assessment; *NIHSS* National Institute of Health Stroke Scale; *mRS* Modified Rankin Scale; *SCQ* Self-Administered Comorbidity Questionnaire; *GDS-15* 15-Item Geriatric Depression Scale; *SSQoL* Stroke-Specific Quality of Life scale; *GPS* Global Positioning System; *UAB LSA* University of Alabama at Birmingham Study of Aging Life-Space Assessment; *UJACAS* University of Jyvaskyla Active Ageing Scale; *FES-I* Falls Efficacy Scale-International

The following characteristics will be assessed at T_0_ and at all follow-up visits (T_1–3_): body weight, financial hardship, residential area (urban/suburban/rural), housing situation (type of housing, floor and availability of an elevator), living condition (alone or with someone else), social support (regular company when going outdoors and for errands) [50] and instrumental activities of daily living (IADLs) [51].

At T_0_ and at T_3_ all subjects will undergo a clinical-neurological examination and the following characteristics will be assessed: stroke severity (National Institutes of Health Stroke Scale; NIHSS) [52], level of functional independence (mRS) [45, 53] comorbidities (Self-Administered Comorbidity Questionnaire; SCQ) [54, 55] and depressive symptoms (Geriatric Depression Scale; GDS-15) [56, 57]. Health-related quality of life will be assessed using the Stroke-Specific Quality of Life scale (SS-QoL); total score as well as subscores will be calculated [58, 59]. Use of centrally acting drugs, vision problems, and hearing problems will also be assessed by self-report.

#### Mobility

Assessments of mobility (the primary outcome of this study) will take place at T_0_ and at all follow-up visits (T_1–3_) (Table 1). In short, walking ability and perceived mobility limitation will be assessed by self-report. LEPF will be assessed by a battery of tests (including quantitative gait analysis, tests of lower limb muscle power, balance tests and functional tests) in a given order at the study centre. All tests will be performed by a trained assessor. Life-space will be assessed by GPS and by self-report questionnaire. Mobility assessments will be complemented by an evaluation of reasons for going outdoors, transportation use and need for assistance by using a questionnaire tool that is based on digital maps.

#### Walking ability and mobility limitation

Pre-stroke (T_0_) and current (T_0–3_) walking ability (no walking aid, cane or rollator) [60] and perceived pre-stroke (T_0_) and current (T_0–3_) mobility limitations (difficulties in walking and climbing stairs) [61] will be assessed by self-report.

#### Quantitative gait analysis

Quantitative gait analysis will be performed by using a pressure-sensitive electronic walkway (GAITRite, Platinum version, active length 972 cm, CIR System Inc., Franklin, NJ, USA) [62–64] and body-worn inertial sensors (Physilog 5, Gait Up SA, Lausanne, Switzerland) [65]. Temporal and spatial gait parameters (e.g. walking speed, cadence, stride time variability, stance time variability, double support time variability, step length, stride length asymmetry, step width variability, stride length variability, and stride time variability) will be derived from a walk on the GAITRite walkway [66, 67]. Toe clearance, as a potential indicator of tripping risk [68], will be derived from the body-worn sensor data [69]. Participants will perform two additional 10 m walks on the walkway under dual-task conditions (working memory task with serial subtraction and verbal fluency task of naming animals) [70, 71]. Participants will be allowed to use their usual walking aid during the walking tests and they always walk at their habitual, comfortable walking speed.

#### Lower limb muscle power

Lower limb muscle power (the product of the strength and velocity of movement) will be assessed in a seated position by using a leg press device that allows testing clinical populations with motor impairments safely (DD System Elite, Dynamic Devices AG, Zürich, Switzerland). As a measure of muscle power, the maximum rate of force development during a concentric action (i.e. a simulated chair-rise) will be used [72]. The device will allow us to measure both legs separately and to quantify the potential asymmetry between legs as a potential risk factor for falls [73]. Studies suggest that leg power is even more predictive of general functional performance of older adults than leg strength [72, 74, 75]. Tests will be performed after a period of familiarisation and warm-up. It has been shown in various studies that testing of muscle power (and even ballistic power training) [76, 77] can be performed safely in patients with stroke [76, 78], in other clinical populations [79], and in mobility-limited [72], and healthy older adults [75, 80].

#### Balance

Balance will be assessed by using a force platform (Leonardo Mechanograph, Novotec Medical GmbH, Pforzheim, Germany) [81]. The following parameters of postural sway while standing quietly in an upright position with knees slightly flexed (~ 10°), hands at the side and gaze straight ahead for 10 s in various foot positions (side-by-side, semi-tandem, and tandem position) will be used: 95% ellipse sway area, and path length [82, 83].

#### Functional tests

The following two functional tests will be performed: 5 times sit-to stand (on the Leonardo Mechanograph force platform), a general test of lower body strength [84, 85] and timed up-and-go test, a measure of general mobility [86–88].

#### Life-space

Immediately after each study centre visit, participants’ location will be continuously recorded by GPS (uTrail, CDD Ltd., Athens, Greece) over a 1-week period. A previously suggested approach to derive an area-related summary measure of life-space (Standard Deviational Ellipse) will be used [89, 90]. Additionally, the University of Alabama at Birmingham (UAB) Study of Aging Life-space Assessment (LSA) questionnaire will be used. The UAB-LSA assesses the extent of an individual’s movement within the past 4 weeks, categorized into 5 spatial levels, ranging from the participant’s bedroom to places outside the participants’ home town, by self-report [10].

#### Reasons for going outdoors, transportation use and need for assistance

In order to collect additional qualitative information on visited locations, including purpose (within the 6 categories work, groceries, social contacts, medical institutions, culture/religion/education and free time), visit frequency, transportation mode, assistance needed, and distances covered, a questionnaire tool that is based on digital maps and usually referred to as ‘Public Participation Geographic Information System’ (PPGIS) [91] or ‘SoftGIS’ [41, 42] will be used (T_0–3_). Furthermore, pre-stroke (T_0_) and current (T_0–3_) availability and usage of a private car will be assessed by self-report.

#### Further measures

##### Habitual physical activity

Physical activity will be assessed at T_0–3_ by using a wrist-worn triaxial accelerometer (GeneActiv, Activinsights Ltd., Kimbolton, UK) [92]. Participants will be asked to wear the bracelet continuously over a 1-week period.

##### Active ageing

Furthermore, “active ageing” will be assessed at T_0–3_ by self-report by using the University of Jyvaskyla Active Ageing Scale [93]. This scale intends to quantify “the striving for elements of well-being through activities relating to a person’s goals, functional capacities and opportunities” [93] (p. 1003).

##### Falls

Falls are a known consequence of mobility problems in patients after stroke [94, 95]. The following fall-related measures will therefore complement the assessments: pre-stroke (T_0_) and current (T_0–3_) frequency of falls (3-month recall) [96], and fall-related self-efficacy (Falls Efficacy Scale–International Version) (T_0–3_) [97].

##### Retinal vessel analysis

Retinal vessel analysis is a non-invasive technique that allows examination of the retinal microcirculation [98]. Retinal vessels share common functional, morphological and embryological characteristics with the cerebrovascular bed. They represent a part of the microvasculature that is affected early in the process of cerebrovascular small vessel disease [99–101]. In the population-based Rotterdam Study, for example, wider retinal venular diameters were associated with increased risk for stroke and cerebral infarction [95]. Retinal vessel diameters will be measured at T_1_ and T_3_. The Static Retinal Vessel Analyzer (SVA-T, Imedos Systems UG, Jena, Germany) will be used to take three images of each eye from each participant with a fundus camera (Topcon TRC NW8), allowing a non-invasive assessment of retinal vessel diameters without mydriasis. Images will be analysed by a semi-automated software (Visualis 2.8, Imedos Systems UG) at an angle of 45° with the optic disc at centre. Retinal vessel diameters are measured in ring zones 0.5–1 disc-diameter away from the optic disc margin. Central retinal arteriolar equivalent (CRAE) and central retinal venular equivalent (CRVE) are averaged by the Hubbard formula [102].

##### Medical events

Potential medical events (e.g. recurrent strokes), other relevant medical issues, as well as medication intake will be recorded (T_0–3_).

### Ethical considerations

The study is fully observational. The performed measurements mainly include or simulate everyday tasks that do not involve an increased cardiovascular or musculoskeletal risk compared to everyday activity. All performance tests have been used safely in previous studies with chronically diseased participants (including stroke) [60, 103] and will be conducted by specifically trained assessors. The assessor will constantly supervise the participant and accompany the participant during tests with a potential risk of falling. Data generation, transmission, storage and analysis of health-related personal data will follow the current Swiss legal requirements for data protection.

### Statistical analysis

Patients’ characteristics will be analysed descriptively. Measures of mobility (LEPF and life-space) will be analysed (descriptive statistics and 95% confidence intervals) for the total sample and stratified by predefined subgroups (based on demographic characteristics and stroke severity). Linear mixed effects models will be used to model the trajectories of mobility measures for the total sample and for predefined subgroups [104, 105]. Time will be modelled as a discrete variable indicating the time points (T_0_, T_1_, T_2_, T_3_). Comparison of trajectories between subgroups will be assessed using likelihood ratio tests [106]. Tukey post-hoc tests will be applied to compare subgroups at specific time points. Diagnostic residual plots will be applied to assess the model fit. Measures of mobility will be transformed (e.g., log) in the case of inadequate model fits.

As an exploratory analysis, growth mixture models (GMMs) will be used to identify relevant subgroups with different trajectories [104, 107–109]. GMMs identify multiple latent classes that have similar trajectories over time. The number of latent classes to be extracted must be set in advance. We will fit GMMs with two to five classes. The model selection will be done based on the lowest value of the Akaike Information Criterion (AIC) and lowest value of the Bayesian Information Criterion (BIC) [108]. After identifying relevant subgroups with different trajectories using GMMs, multinomial logistic regression models will be used to identify predictors of group-membership [110].

As a secondary analysis, we will use linear mixed effect models to test whether changes in LEPF parameters are associated with changes in life-space [111]. Specifically, the changes in life-space between each time point serves as the dependent variable and the changes in LEPF parameters serve as predictors.

Data from the map-based tool including reasons for going outdoors, transportation use, distances covered and assistance needed will be analysed descriptively (frequencies and proportions with 95% confidence intervals) by subgroups of age, sex, stroke severity, housing/living situation, and residential area.

We set the significance level at 5% and will use two-sided tests throughout our analyses.

### Sample size calculation

We used simulations to estimate the required sample size for a linear mixed effects model assessing the trajectory of 10 m habitual walking speed over 4 time points [112]. Specifically, we powered the analysis to detect an overall trend over time as well as clinically significant differences of 0.14 m/s between each adjacent time point [13, 113, 114]. Based on prior studies, the standard deviation of the walking speed was assumed to be 0.3 m/s at each time point [13, 66, 113–116]. We found no information in the literature about the correlation of walking speed within each subject between time points. Accordingly, we performed multiple simulations for correlations between time points of 0.2, 0.5, 0.6, and 0.7, while keeping all other parameters fixed. For each assumed correlation, a simulation with 5000 repetitions to assure robust estimation was performed to estimate the required sample size. All hypothesis tests were two-sided with a significance level of 5% while the statistical power was set at 80%. We inflated the required sample sizes to adjust for an anticipated dropout rate of 5% between each time point.

**Fig. 2:**
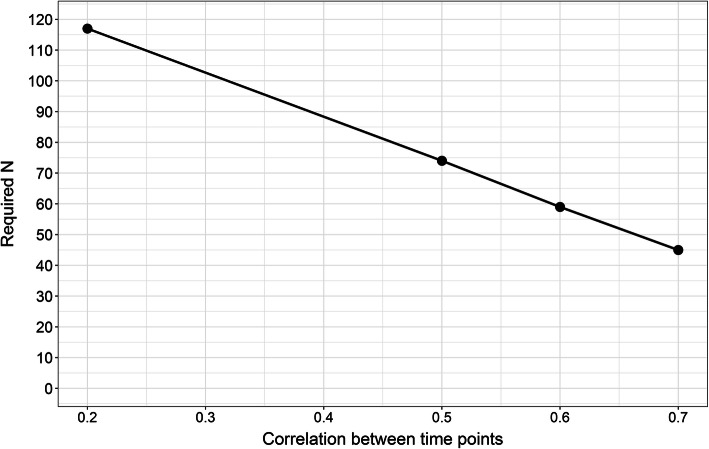
Required sample sizes for a statistical power of 80% assuming various correlations between time points within subjects

Assuming a correlation of 0.2 of walking speed between time points, 117 subjects were needed to achieve the required power of 80%. The corresponding sample sizes were 74, 59 and 45 for correlations of 0.5, 0.6 and 0.7, respectively (Fig. 2). We think that a correlation of at least 0.6 is plausible for these data. Thus, we decided to at least recruit 59 subjects.

## Discussion

Regaining mobility is a primary rehabilitation goal of patients after stroke and a comprehensive and detailed knowledge of functional deficits and their recovery patterns will enable the planning of targeted and correctly timed rehabilitation measures ranging from targeted exercise to the provision of aids or adaptations of the built environment. The integration of both aspects of mobility, LEPF and life-space, will provide the opportunity to define individualized and motivating patient-oriented goals and potentially booster the efforts of therapists by self-encouragement of the patient, potentially leading to an increase of quality of life and participation following stroke. The low cost and high availability of GPS and portable sensor technology as well as the fact that measurements mostly rely on unsupervised procedures may facilitate the future use of these measures by researchers and clinicians.

## Data Availability

After completion of the project, data sets will be made publicly available.

## Abbreviations

ADL: Activities of daily life
GMMs: Growth mixture models
GPS: Global positioning system
LSA: Life-space assessment
MoCA: Montreal cognitive assessment
mRS: Modified Rankin scale
SS-QoL: Stroke-specific quality of life
UAB: University of Alabama in Birmingham

## References

[1] Petty GW, Brown RD, Whisnant JP, Sicks JD, O'Fallon WM, Wiebers DO (2000). Ischemic stroke subtypes : a population-based study of functional outcome, survival, and recurrence. Stroke.

[2] Rothwell PM, Coull AJ, Silver LE, Fairhead JF, Giles MF, Lovelock CE (2005). Population-based study of event-rate, incidence, case fatality, and mortality for all acute vascular events in all arterial territories (Oxford vascular study). Lancet.

[3] Truelsen T, Piechowski-Jozwiak B, Bonita R, Mathers C, Bogousslavsky J, Boysen G (2006). Stroke incidence and prevalence in Europe: a review of available data. Eur J Neurol.

[4] Bravata DM, Ho SY, Brass LM, Concato J, Scinto J, Meehan TP (2003). Long-term mortality in cerebrovascular disease. Stroke.

[5] Strong K, Mathers C, Bonita R (2007). Preventing stroke: saving lives around the world. Lancet Neurol.

[6] Hankey GJ (2003). Long-term outcome after ischaemic stroke/transient ischaemic attack. Cerebrovasc Dis.

[7] Webber SC, Porter MM, Menec VH (2010). Mobility in older adults: a comprehensive framework. Gerontologist.

[8] Macko RF, Benvenuti F, Stanhope S, Macellari V, Taviani A, Nesi B (2008). Adaptive physical activity improves mobility function and quality of life in chronic hemiparesis. J Rehabil Res Dev.

[9] Guralnik JM, Simonsick EM, Ferrucci L, Glynn RJ, Berkman LF, Blazer DG (1994). A short physical performance battery assessing lower extremity function: association with self-reported disability and prediction of mortality and nursing home admission. J Gerontol.

[10] Baker PS, Bodner EV, Allman RM (2003). Measuring life-space mobility in community-dwelling older adults. J Am Geriatr Soc.

[11] Lee KB, Lim SH, Kim KH, Kim KJ, Kim YR, Chang WN (2015). Six-month functional recovery of stroke patients: a multi-time-point study. Int J Rehabil Res.

[12] Combs SA, Dugan EL, Passmore M, Riesner C, Whipker D, Yingling E (2010). Balance, balance confidence, and health-related quality of life in persons with chronic stroke after body weight-supported treadmill training. Arch Phys Med Rehab.

[13] Fulk GD, Ludwig M, Dunning K, Golden S, Boyne P, West T (2011). Estimating clinically important change in gait speed in people with stroke undergoing outpatient rehabilitation. J Neurol Phys Ther.

[14] Ilunga Tshiswaka D, Bennett C, Franklin C (2018). Effects of walking trainings on walking function among stroke survivors: a systematic review. Int J Rehabil Res.

[15] Blennerhassett JM, Dite W, Ramage ER, Richmond ME (2012). Changes in balance and walking from stroke rehabilitation to the community: a follow-up observational study. Arch Phys Med Rehabil.

[16] Ezeugwu VE, Garga N, Manns PJ (2017). Reducing sedentary behaviour after stroke: perspectives of ambulatory individuals with stroke. Disabil Rehabil.

[17] Scherbakov N, von Haehling S, Anker SD, Dirnagl U, Doehner W (2013). Stroke induced sarcopenia: muscle wasting and disability after stroke. Int J Cardiol.

[18] Goh HT, Nadarajah M, Hamzah NB, Varadan P, Tan MP (2016). Falls and fear of falling after stroke: a case-control study. Pm&R.

[19] Carod-Artal J, Egido JA, Gonzalez JL, Varela de Seijas E (2000). Quality of life among stroke survivors evaluated 1 year after stroke: experience of a stroke unit. Stroke.

[20] Moreland JD, Depaul VG, Dehueck AL, Pagliuso SA, Yip DW, Pollock BJ (2009). Needs assessment of individuals with stroke after discharge from hospital stratified by acute functional Independence measure score. Disabil Rehabil.

[21] Hankey GJ (2003). Long-term outcome after ischaemic stroke/transient ischaemic attack. Cerebrovasc Dis.

[22] Ilunga Tshiswaka D, Bennett C, Franklin C (2017). Effects of walking trainings on walking function among stroke survivors: a systematic review. Int J Rehabil Res.

[23] Hendrickson J, Patterson KK, Inness EL, McIlroy WE, Mansfield A (2014). Relationship between asymmetry of quiet standing balance control and walking post-stroke. Gait Posture.

[24] Szopa A, Domagalska-Szopa M, Lasek-Bal A, Zak A (2017). The link between weight shift asymmetry and gait disturbances in chronic hemiparetic stroke patients. Clin Interv Aging.

[25] Tyson SF, Kent RM (2013). Effects of an ankle-foot orthosis on balance and walking after stroke: a systematic review and pooled meta-analysis. Arch Phys Med Rehabil.

[26] Duncan P, Studenski S, Richards L, Gollub S, Lai SM, Reker D (2003). Randomized clinical trial of therapeutic exercise in subacute stroke. Stroke.

[27] Han P, Zhang W, Kang L, Ma Y, Fu L, Jia L (2017). Clinical evidence of exercise benefits for stroke. Adv Exp Med Biol.

[28] Nudo RJ (2003). Functional and structural plasticity in motor cortex: implications for stroke recovery. Phys Med Rehabil Clin N Am.

[29] Salter K, Jutai J, Hartley M, Foley N, Bhogal S, Bayona N (2006). Impact of early vs delayed admission to rehabilitation on functional outcomes in persons with stroke. J Rehabil Med.

[30] Yao M, Chen J, Jing J, Sheng H, Tan X, Jin J (2017). Defining the rehabilitation adherence curve and adherence phases of stroke patients: an observational study. Patient Prefer Adher.

[31] Jurkiewicz MT, Marzolini S, Oh P (2011). Adherence to a home-based exercise program for individuals after stroke. Top Stroke Rehabil.

[32] Lord SE, McPherson K, McNaughton HK, Rochester L, Weatherall M (2004). Community ambulation after stroke: how important and obtainable is it and what measures appear predictive?. Arch Phys Med Rehab.

[33] Tung JY, Rose RV, Gammada E, Lam I, Roy EA, Black SE (2014). Measuring life space in older adults with mild-to-moderate Alzheimer's disease using mobile phone GPS. Gerontology.

[34] Portegijs E, Rantakokko M, Viljanen A, Sipila S, Rantanen T (2016). Identification of older people at risk of ADL disability using the life-space assessment: a longitudinal cohort study. J Am Med Dir Assoc.

[35] Xue QL, Fried LP, Glass TA, Laffan A, Chaves PH (2008). Life-space constriction, development of frailty, and the competing risk of mortality: the Women's health and aging study I. Am J Epidemiol.

[36] Lo AX, Brown CJ, Sawyer P, Kennedy RE, Allman RM (2014). Life-space mobility declines associated with incident falls and fractures. J Am Geriatr Soc.

[37] Sheppard KD, Sawyer P, Ritchie CS, Allman RM, Brown CJ (2013). Life-space mobility predicts nursing home admission over 6 years. J Aging Health.

[38] Boyle PA, Buchman AS, Barnes LL, James BD, Bennett DA (2010). Association between life space and risk of mortality in advanced age. J Am Geriatr Soc.

[39] Harada K, Lee S, Lee S, Bae S, Harada K, Suzuki T (2017). Objectively-measured outdoor time and physical and psychological function among older adults. Geriatr Gerontol Int.

[40] Hirsch JA, Winters M, Ashe MC, Clarke P, McKay H (2016). Destinations that older adults experience within their GPS activity spaces relation to objectively measured physical activity. Environ Behav.

[41] Rantanen H, Kahila M (2009). The SoftGIS approach to local knowledge. J Environ Manag.

[42] Kytta AM, Broberg AK, Kahila MH (2012). Urban environment and children's active lifestyle: softGIS revealing children's behavioral patterns and meaningful places. Am J Health Promot.

[43] Hinrichs T, Keskinen KE, Pavelka B, Eronen J, Schmidt-Trucksass A, Rantanen T (2019). Perception of parks and trails as mobility facilitators and transportation walking in older adults: a study using digital geographical maps. Aging Clin Exp Res.

[44] Yang YN, Kim BR, Uhm KE, Kim SJ, Lee S, Oh-Park M (2017). Life space assessment in stroke patients. Ann Rehabil Med.

[45] van Swieten JC, Koudstaal PJ, Visser MC, Schouten HJ, van Gijn J (1988). Interobserver agreement for the assessment of handicap in stroke patients. Stroke.

[46] Berger K, Weltermann B, Kolominsky-Rabas P, Meves S, Heuschmann P, Bohner J (1999). The reliability of stroke scales. The german version of NIHSS, ESS and Rankin scales. Fortschr Neurol Psychiatr.

[47] Dong YH, Xu J, Chan BPL, Seet RCS, Venketasubramanian N, Teoh HL (2016). The Montreal cognitive assessment is superior to national institute of neurological disease and stroke-Canadian stroke network 5-minute protocol in predicting vascular cognitive impairment at 1 year. BMC Neurol.

[48] Nasreddine ZS, Phillips NA, Bedirian V, Charbonneau S, Whitehead V, Collin I (2005). The Montreal cognitive assessment, MoCA: a brief screening tool for mild cognitive impairment. J Am Geriatr Soc.

[49] Brott T, Adams HP, Olinger CP, Marler JR, Barsan WG, Biller J (1989). Measurements of acute cerebral infarction: a clinical examination scale. Stroke.

[50] Rantanen T, Portegijs E, Viljanen A, Eronen J, Saajanaho M, Tsai LT (2012). Individual and environmental factors underlying life space of older people - study protocol and design of a cohort study on life-space mobility in old age (LISPE). BMC Public Health.

[51] Bundesamt für Statistik. Die Schweizerische Gesundheitsbefragung 2017. 2018.

[52] Brott T, Adams HP, Olinger CP, Marler JR, Barsan WG, Biller J (1989). Measurements of acute cerebral infarction - a clinical examination scale. Stroke.

[53] Vanswieten JC, Koudstaal PJ, Visser MC, Schouten HJA, Vangijn J (1988). Interobserver agreement for the assessment of handicap in stroke patients. Stroke.

[54] Streibelt M, Schmidt C, Brünger M, Spyra K (2012). Komorbidität im Patientenurteil–geht das?. Orthopade.

[55] Sangha O, Stucki G, Liang MH, Fossel AH, Katz JN (2003). The self-administered comorbidity questionnaire: a new method to assess comorbidity for clinical and health services research. Arthritis Rheum.

[56] Yesavage JA, Brink TL, Rose TL, Lum O, Huang V, Adey M (1982). Development and validation of a geriatric depression screening scale: a preliminary report. J Psychiatr Res.

[57] Baumgartner JS, Jahn R, Friedrich F, Alexandrowicz RW, Wancata J (2019). Criterion validity of the 15-item geriatric depression scale in the Austrian population. Psychiatr Prax.

[58] Williams LS, Weinberger M, Harris LE, Clark DO, Biller J (1999). Development of a stroke-specific quality of life scale. Stroke.

[59] Ewert T, Stucki G (2007). Validity of the SS-QOL in Germany and in survivors of hemorrhagic or ischemic stroke. Neurorehabil Neural Repair.

[60] Hinrichs T, Moschny A, Brach M, Wilm S, Klaassen-Mielke R, Trampisch M (2011). Effects of an exercise programme for chronically ill and mobility-restricted elderly with structured support by the general practitioner's practice (HOMEfit) - study protocol of a randomised controlled trial. Trials.

[61] Rasinaho M, Hirvensalo M, Leinonen R, Lintunen T, Rantanen T (2007). Motives for and barriers to physical, activity among older adults with mobility limitations. J Aging Phys Activ.

[62] McDonough AL, Batavia M, Chen FC, Kwon S, Ziai J (2001). The validity and reliability of the GAITRite system's measurements: a preliminary evaluation. Arch Phys Med Rehabil.

[63] Kuys SS, Brauer SG, Ada L (2011). Test-retest reliability of the GAITRite system in people with stroke undergoing rehabilitation. Disabil Rehabil.

[64] Wong JS, Jasani H, Poon V, Inness EL, McIlroy WE, Mansfield A (2014). Inter- and intra-rater reliability of the GAITRite system among individuals with sub-acute stroke. Gait Posture.

[65] Schwameder H, Andress M, Graf E, Strutzenberger G. Validation of an IMU-System (Gait-Up) to identify gait parameters in normal and induced limping walking conditions. In: ISBS-Conference Proceedings Archive. 33rd International Conference on Biomechanics in Sport. 2015. https://ojs.ub.uni-konstanz.de/cpa/article/view/6495. Accessed 1 Sept 2020.

[66] Choi W, Han D, Kim J, Lee S (2017). Whole-body vibration combined with treadmill training improves walking performance in post-stroke patients: a randomized controlled trial. Med Sci Monit.

[67] Brach JS, Berlin J, VanSwearingen J, Newman A, Studenski S (2005). Too much or too little step width variability is associated with a fall history only in older persons who walk at or near normal gait speed. J Am Geriatr Soc.

[68] Hamacher D, Hamacher D, Schega L (2014). Towards the importance of minimum toe clearance in level ground walking in a healthy elderly population. Gait Posture.

[69] Washabaugh EP, Kalyanaraman T, Adamczyk PG, Claflin ES, Krishnan C (2017). Validity and repeatability of inertial measurement units for measuring gait parameters. Gait Posture.

[70] Bridenbaugh SA, Kressig RW (2015). Motor cognitive dual tasking: early detection of gait impairment, fall risk and cognitive decline. Z Gerontol Geriatr.

[71] Bridenbaugh SA, Kressig RW (2011). Laboratory review: the role of gait analysis in seniors' mobility and fall prevention. Gerontology.

[72] Bean JF, Kiely DK, Herman S, Leveille SG, Mizer K, Frontera WR (2002). The relationship between leg power and physical performance in mobility-limited older people. J Am Geriatr Soc.

[73] Chon J, Kim HS, Lee JH, Yoo SD, Yun DH, Kim DH (2018). Association between asymmetry in knee extension strength and balance in a community-dwelling elderly population: a cross-sectional analysis. Ann Rehabil Med.

[74] Mayson DJ, Kiely DK, LaRose SI, Bean JF (2008). Leg strength or velocity of movement: which is more influential on the balance of mobility limited elders?. Am J Phys Med Rehab.

[75] Bean JF, Leveille SG, Kiely DK, Bandinelli S, Guralnik JM, Ferrucci L (2003). A comparison of leg power and leg strength within the InCHIANTI study: which influences mobility more?. J Gerontol A Biol Sci Med Sci.

[76] Hendrey G, Clark RA, Holland AE, Mentiplay BF, Davis C, Windfeld-Lund C (2018). Feasibility of ballistic strength training in subacute stroke: a randomized, controlled, Assessor-blinded pilot study. Arch Phys Med Rehabil.

[77] Aaron SE, Hunnicutt JL, Embry AE, Bowden MG, Gregory CM (2017). POWER training in chronic stroke individuals: differences between responders and nonresponders. Top Stroke Rehabil.

[78] Kostka J, Niwald M, Guligowska A, Kostka T, Miller E (2019). Muscle power, contraction velocity and functional performance after stroke. Brain Behav.

[79] Portegijs E, Sipila S, Rantanen T, Lamb SE (2008). Leg extension power deficit and mobility limitation in women recovering from hip fracture. Am J Phys Med Rehabil.

[80] Portegijs E, Sipila S, Alen M, Kaprio J, Koskenvuo M, Tiainen K (2005). Leg extension power asymmetry and mobility limitation in healthy older women. Arch Phys Med Rehabil.

[81] Taani MH, Kovach CR, Buehring B (2017). Muscle Mechanography: a novel method to measure muscle function in older adults. Res Gerontol Nurs.

[82] Horak F, King L, Mancini M (2015). Role of body-worn movement monitor technology for balance and gait rehabilitation. Phys Ther.

[83] Remaud A, Boyas S, Caron GA, Bilodeau M (2012). Attentional demands associated with postural control depend on task difficulty and visual condition. J Motor Behav.

[84] Mancini M, Smulders K, Cohen RG, Horak FB, Giladi N, Nutt JG (2017). The clinical significance of freezing while turning in Parkinson's disease. Neuroscience.

[85] Mancini M, Schlueter H, El-Gohary M, Mattek N, Duncan C, Kaye J (2016). Continuous monitoring of turning mobility and its association to falls and cognitive function: a pilot study. J Gerontol A Biol Sci Med Sci.

[86] Podsiadlo D, Richardson S (1991). The timed "up & go" - a test of basic functional mobility for frail elderly persons. J Am Geriatr Soc.

[87] Coulthard JT, Treen TT, Oates AR, Lanovaz JL (2015). Evaluation of an inertial sensor system for analysis of timed-up-and-go under dual-task demands. Gait Posture.

[88] Rikli RE, Jones CJ (1999). Development and validation of a functional fitness test for community-residing older adults. J Aging Phys Act.

[89] Hirsch JA, Winters M, Clarke P, McKay H (2014). Generating GPS activity spaces that shed light upon the mobility habits of older adults: a descriptive analysis. Int J Health Geogr.

[90] Münch M, Weibel R, Sofios A, Huang H, Infanger D, Portegijs E (2019). MOBIlity assessment with modern TEChnology in older patients' real-life by the general practitioner: the MOBITEC-GP study protocol. BMC Public Health.

[91] Laatikainen TE, Broberg A, Kytta M (2017). The physical environment of positive places: exploring differences between age groups. Prev Med.

[92] Esliger DW, Rowlands AV, Hurst TL, Catt M, Murray P, Eston RG (2011). Validation of the GENEA accelerometer. Med Sci Sports Exerc.

[93] Rantanen T, Portegijs E, Kokko K, Rantakokko M, Tormakangas T, Saajanaho M (2019). Developing an assessment method of active aging: University of Jyvaskyla Active Aging Scale. J Aging Health.

[94] van der Kooi E, Schiemanck SK, Nollet F, Kwakkel G, Meijer JW, van de Port I (2017). Falls are associated with lower self-reported functional status in patients after stroke. Arch Phys Med Rehab.

[95] Ng MM, Hill KD, Batchelor F, Burton E (2017). Factors predicting falls and mobility outcomes in patients with stroke returning home after rehabilitation who are at risk of falling. Arch Phys Med Rehab.

[96] Lamb SE, Jorstad-Stein EC, Hauer K, Becker C (2005). Prevention of falls network E, outcomes consensus G. development of a common outcome data set for fall injury prevention trials: the prevention of falls network Europe consensus. J Am Geriatr Soc.

[97] Dias N, Kempen GI, Todd CJ, Beyer N, Freiberger E, Piot-Ziegler C (2006). The German version of the falls efficacy scale-international version (FES-I). Z Gerontol Geriatr.

[98] Liew G, Wang JJ, Mitchell P, Wong TY (2008). Retinal vascular imaging: a new tool in microvascular disease research. Circ Cardiovasc Imaging.

[99] Ikram MK, de Jong FJ, Bos MJ, Vingerling JR, Hofman A, Koudstaal PJ (2006). Retinal vessel diameters and risk of stroke: the Rotterdam study. Neurology..

[100] Hughes AD, Falaschetti E, Witt N, Wijetunge S, Thom SA, Tillin T (2016). Association of Retinopathy and Retinal Microvascular Abnormalities with Stroke and cerebrovascular disease. Stroke.

[101] Wu HQ, Wu H, Shi LL, Yu LY, Wang LY, Chen YL (2017). The association between retinal vasculature changes and stroke: a literature review and meta-analysis. Int J Ophthalmol Chi.

[102] Hubbard LD, Brothers RJ, King WN, Clegg LX, Klein R, Cooper LS (1999). Methods for evaluation of retinal microvascular abnormalities associated with hypertension/sclerosis in the atherosclerosis risk in communities study. Ophthalmology.

[103] Hinrichs T, Bucchi C, Brach M, Wilm S, Endres HG, Burghaus I (2009). Feasibility of a multidimensional home-based exercise programme for the elderly with structured support given by the general practitioner's surgery: study protocol of a single arm trial preparing an RCT [ISRCTN58562962]. BMC Geriatr.

[104] West BT, Welch KB, Galecki AT (2014). Linear mixed models : a practical guide using statistical software. Second edition. Ed.

[105] Pinheiro JC, Bates DM (2000). Mixed effects models in S and S-PLUS.

[106] Long JD. Longitudinal data analysis for the behavioral sciences using R. Thousand Oaks, Calif.: SAGE; 2012. xxii, 542 p. p.

[107] Martin DP, von Oertzen T (2015). Growth mixture models outperform simpler clustering algorithms when detecting longitudinal heterogeneity, even with small sample sizes. Struct Equ Model.

[108] Ram N, Grimm KJ (2009). Growth mixture Modeling: a method for identifying differences in longitudinal change among unobserved groups. Int J Behav Dev.

[109] Nylund KL, Asparoutiov T, Muthen BO (2007). Deciding on the number of classes in latent class analysis and growth mixture modeling: a Monte Carlo simulation study. Struct Equ Model.

[110] Hosmer DW, Lemeshow S, Sturdivant RX (2013). Applied logistic regression. 3rd ed. ed.

[111] Twisk JWR. Applied longitudinal data analysis for epidemiology : a practical guide. Second Edition. ed. Cambridge ; New York: Cambridge University Press; 2013. xiv, 321 pages p.

[112] Arnold BF, Hogan DR, Colford JM, Hubbard AE (2011). Simulation methods to estimate design power: an overview for applied research. BMC Med Res Methodol.

[113] Perera S, Mody SH, Woodman RC, Studenski SA (2006). Meaningful change and responsiveness in common physical performance measures in older adults. J Am Geriatr Soc.

[114] Tilson JK, Sullivan KJ, Cen SY, Rose DK, Koradia CH, Azen SP (2010). Meaningful gait speed improvement during the first 60 days poststroke: minimal clinically important difference. Phys Ther.

[115] Mackay-Lyons M, McDonald A, Matheson J, Eskes G, Klus MA (2013). Dual effects of body-weight supported treadmill training on cardiovascular fitness and walking ability early after stroke: a randomized controlled trial. Neurorehabil Neural Repair.

[116] Flansbjer UB, Holmback AM, Downham D, Patten C, Lexell J (2005). Reliability of gait performance tests in men and women with hemiparesis after stroke. J Rehabil Med.

